# Establishment of a bead‐based duplex assay for the simultaneous quantitative detection of Neuropilin‐1 and Neuropilin‐2 using xMAP technology and its clinical application

**DOI:** 10.1002/jcla.22850

**Published:** 2019-02-13

**Authors:** Zi‐Lan Huang, Pei‐Pei Meng, Yun Yang, Sheng‐Yu Wang, Xiu‐fang Zhang, Fang‐Hong Luo, Jiang‐Hua Yan, Ting Wu

**Affiliations:** ^1^ Cancer Research Center, Medical College Xiamen University Xiamen China; ^2^ Department of Pediatrics Xiang’an Hospital of Xiamen University Xiamen China

**Keywords:** bead‐based immunoassay, duplex flow cytometry, Neuropilins, xMAP technology

## Abstract

**Background:**

Neuropilins (Nrps) are a new type of broad‐spectrum tumor marker. Currently, a method for accurate simultaneous quantification of Nrps is not available. We aimed to develop a bead‐based and duplexed flow cytometric assay that could be used for accurate and simultaneous quantification of Nrp1 and Nrp2 for scientific research or clinical diagnosis.

**Methods:**

We coupled anti‐human Nrp1‐11# mAb and anti‐human Nrp2‐C3 mAb to magnetic beads 18# and 25#, respectively. Capturing antibodies and detecting antibodies were then combined to detect Nrps by a bead‐based Luminex assay, which was subsequently applied to quantify Nrps in clinical serum samples.

**Results:**

The results showed that the detection value of Nrps ranged from 10 to 100 000 pg/mL for Nrp1 and from 25 to 100 000 pg/mL for Nrp2. The detection sensitivity reached 10 pg/mL for Nrp1 and 24.8 pg/mL for Nrp2. Intra‐assay variances ranged from 1.0% to 2.6% for Nrp1 and from 2.9% to 4.0% for Nrp2, and interassay variances ranged from 1.5% to 6.4% for Nrp1 and from 4.2% to 8.1% for Nrp2. The Nrp1 and Nrp2 recoveries were 96.6%‐103.6% and 95.6%‐102.3%, respectively. Irrelevant antigens had no interference in the paired‐detection system, and the mean fluorescence intensity (MFI) values were stable for months.

**Conclusion:**

A bead‐based, duplexed flow cytometric assay (xMAP^®^ technology) was developed to detect Nrp1 and Nrp2. The assay provided rapid, high‐throughput results and was much more sensitive, specific, reproducible, and stable than existing assays. In addition, this assay could be applied in early‐stage cancer screening, tumor malignancy analysis, and prognosis assessment.

## 1 INTRODUCTION

Tumor markers play an important role in clinical diagnosis and tumor treatment. The detection of tumor markers in the blood or body fluids is useful not only for general assessments, early diagnosis, auxiliary diagnosis, differential diagnosis, and clinical staging of tumors but also for monitoring curative effects and predicting prognosis.

Neuropilins (Nrps) are multifunctional coreceptors for class 3 semaphorins, playing critical roles in axonal guidance,^1^, ^2^ and for members of the vascular endothelial growth factor (VEGF) family in angiogenesis.^3^ Considering Nrp1 and Nrp2, which are two types of Nrps, Nrp1 is essential for neuronal and cardiovascular development, whereas Nrp2 plays key roles in neuronal patterning and lymphangiogenesis. Furthermore, Nrps are highly expressed in diverse human tumors and have been implicated in tumor growth and vascularization.^4^

The liquid‐phase chip, also known as a suspension array, flow cytometry or a fluorescence technique, is a new biochip technology platform based on xMAP Luminex Multi‐Analyte (Luminex 100™) technology from the United States. The technology involves an antigen‐antibody, enzyme‐substrate, ligand‐receptor, or nucleic acid hybridization binding reaction, which is carried out on different fluorescent‐encoded microspheres, and qualitative and quantitative results are obtained by detecting the respective coding of microspheres and fluorescence signals of reactions by red and green laser beams. As many as 100 different biological reactions can be completed simultaneously, thus representing a new generation of high‐throughput molecular diagnostic technology platforms.^5^, ^6^

Liquid chip technology is faster, much more sensitive and flexible, and has a wider range of detection than other immunoassay methods, and its prominent advantage is that it can be simultaneously used in qualitative and quantitative assays for a variety of different target molecules in the same sample.^7^, ^8^, ^9^, ^10^, ^11^

In this study, the double‐antibody‐sandwich immunoassay principle is applied to detect Nrp1 and Nrp2 in human serum by the liquid chip technique, and the dynamic range, sensitivity, cross‐reactivity, intra‐assay and interassay variances, spike recovery, reproducibility, and stability of this developed assay are evaluated. We developed a high‐throughput, combined quantitative detection system for Nrp1 and Nrp2 based on liquid chip technology as a potential new method for the early detection, monitoring, and clinical prognosis prediction of cancer.

## 2 MATERIALS AND METHODS

### 2.1 Reagents

Magnetic beads (18#, Cat. No. MC10018‐01; 25#, Cat. No. MC10025‐01), an xMAP Antibody Coupling Kit (Cat. No. 40‐50016), and a Luminex 200 instrument were purchased from Luminex (Luminex, Austin, TX, USA). A biotin labeling kit (Cat. No. EBLK0002) was purchased from Elabscience (Elabscience, Wuhan, China). Goat anti‐mouse horseradish peroxidase (HRP)‐conjugated secondary antibody, goat anti‐mouse phycoerythrin‐conjugated secondary antibody (IgG‐PE), and streptavidin‐phycoerythrin (SA‐PE) were purchased from Sigma Chemicals Company (St. Louis, MO). O‐phenylenediamine (OPD) was purchased from Sangon (Shanghai, China).

The recombinant protein Nrp1 and the paired‐monoclonal antibodies of Nrp1s and Nrp2s were prepared in‐house according to our previous work. The recombinant protein Nrp2 was kindly provided by Professor Craig W. Vander Kooi (Department of Molecular and Cellular Biochemistry and Center for Structural Biology, University of Kentucky) (Table 1).

**Table 1:** Antibodies, beads, and standards used in the duplex assay

Analyte	Reagent	Clone	Description/bead number
NRP1	Capture Ab	NRP1‐11#	mAb human IgG
	Detection Ab	NRP1‐23#	mAb human IgG
	Standard	**—**	Recombinant protein
	Magnetic bead	**—**	18#
NRP2	Capture Ab	NRP2‐C3	mAb human IgG
	Detection Ab	NRP2‐E4	mAb human IgG
	Standard	**—**	Recombinant protein
	Magnetic bead	**—**	25#

Ab, antibody; mAb, monoclonal antibody.

Human serum samples shown in Table 2 were obtained from the First Affiliated Hospital of Xiamen University and were stored at −20°C before use.

**Table 2:** Resource of human blood serum samples

Serum samples	Number	Source
Normal human serum	100	The First Affiliated Hospital of Xiamen University
Tumor patient serum	45^a^	The First Affiliated Hospital of Xiamen University

a 32 were males, 18 for gastric cancer and 14 for colorectal cancer; 13 were females, eight for gastric cancer and five for colorectal cancer.

### 2.2 Technology

The assays were based on Luminex xMAP technology, a multiplexed sandwich immunoassay technique performed on the surface of 6.5‐μm magnetic polystyrene beads. The following steps are involved.

### 2.3 Bead coupling

To establish the assay, mAbs were independently coupled to magnetic beads. Anti‐human Nrp1‐11# mAb (used as Nrp1‐capturing antibody) was coupled to bead 18#, and anti‐human NRP2‐C3 mAb (used as Nrp2‐capturing antibody) was coupled to bead 25#. An xMAP Antibody Coupling Kit (Cat. No. 40‐50016) was used. The specific steps were as follows: The kit and all reagents were removed from the refrigerator and allowed to equilibrate to room temperature for 20‐30 minutes. The stock microspheres were resuspended on a rotator for 30 seconds. The desired amount of microspheres was dispensed in the reaction tube, and the microspheres were washed with activation buffer three times. The microspheres were activated with sulfo‐*N*‐hydroxysulfosuccinimide (sulfo‐NHS) and EDC solutions and incubated for 20 minutes. The microspheres were then washed with activation buffer three times, and antibodies were added for incubation for 2 hours. The microspheres were washed and resuspended in activation buffer. A total of 150‐200 μg of the respective mAb and 2.5 × 10^6^ beads were used per reaction. After each coupling reaction, the beads were counted to assess the amount of beads recovered and were stored in the dark at 2‐8°C.

### 2.4 Antibody biotinylation

For the bead‐based Luminex assay, each one from the paired antibodies was biotinylated to enable detection of Ab‐B. Anti‐human NRP1‐23#‐B and anti‐human NRP2‐E4‐B were biotinylated. Biotinylation was performed using a biotin labeling kit according to the manufacturer's instructions.

### 2.5 Multiplex assay

Antibody‐coupled beads were diluted in assay buffer (PBS‐BSA solution, 1% BSA) at a concentration of 2000 beads per 50 μL. The bead suspension (50 μL per well) was added to a 96‐well plate. After washing the beads with assay buffer, standards/samples diluted in assay buffer were added to the wells (100 μL per well). Incubation was performed for 2 hours at room temperature, protected from light with continuous gentle shaking, followed by two washing steps. The biotinylated detection antibodies were diluted in assay buffer and added to the wells for incubation for 1 hours with gentle shaking in the dark (100 μL per well). After washing the beads twice, streptavidin‐PE (at 25 μg/mL, 50 μL per well) was added for 30 minutes of incubation. The beads were subsequently washed and resuspended in 100 μL assay buffer and analyzed by the Luminex 200 system.

### 2.6 Sandwich ELISA

To conduct double‐antibody‐sandwich ELISA, 10 μg/mL capturing antibody in coating buffer (0.05 mol/L bicarbonate, pH 9.6) was used for coating the wells of a 96‐well plate overnight at 4°C. Then, the wells of the plate were blocked with 2% bovine serum at 37°C for 1.5 hours and dried by discarding the blocking buffer. Next, Nrp1 and Nrp2 protein samples diluted in PBS at 1 μg/mL followed by double dilution were incubated in a 96‐well plate at 37°C for 2 hours. After washing, goat anti‐mouse HRP‐conjugated secondary antibody was added, and the plate was further incubated at 37°C for 45 minutes. OPD with 0.04% hydrogen peroxide (H_2_O_2_) was added to develop color for 10 minutes, and the optical density (OD) was measured at 490 nm by a microplate reader (model 680, Bio‐Rad, Tokyo, Japan).

### 2.7 Assessment of cross‐reactivity

To analyze cross‐reactivity, single standards containing one of the recombinant proteins (Sema3F, VEGF, CD34, NRP1 or NRP2) at a known concentration were run in the presence of all capturing beads and all biotinylated reporters. The mean fluorescence intensities were detected by the Luminex 200 system.

### 2.8 Assay reproducibility

To determine intra‐assay and interassay variances, three replicates of each sample at three known concentrations (1 ng/mL, 20 ng/mL, 200 ng/mL) were either read in the same batch or read in different batches. The obtained values were compared, and the respective coefficient of variation was calculated by the following formula: coefficient of variation = (standard deviation/average) × 100.

### 2.9 Spike recovery

Ethylene diamine tetraacetic acid (EDTA)‐treated plasma samples were spiked with known amounts of recombinant proteins and analyzed to compare the expected value to the actual value measured in the spiked plasma. This method was used to assess variability due to assay preparation, the interference of substances present in the sample or the sample matrix, and the regression analysis.^12^

### 2.10 Stability

The antibody‐cross‐linked beads, antigen standards, and biotin‐labeled antibodies were kept at 4°C for different numbers of days, and then, their fluorescence values were detected by the Luminex 200 system.

## 3 RESULTS

### 3.1 Confirmation of bead conjugation to capturing antibodies

**Figure 1:**
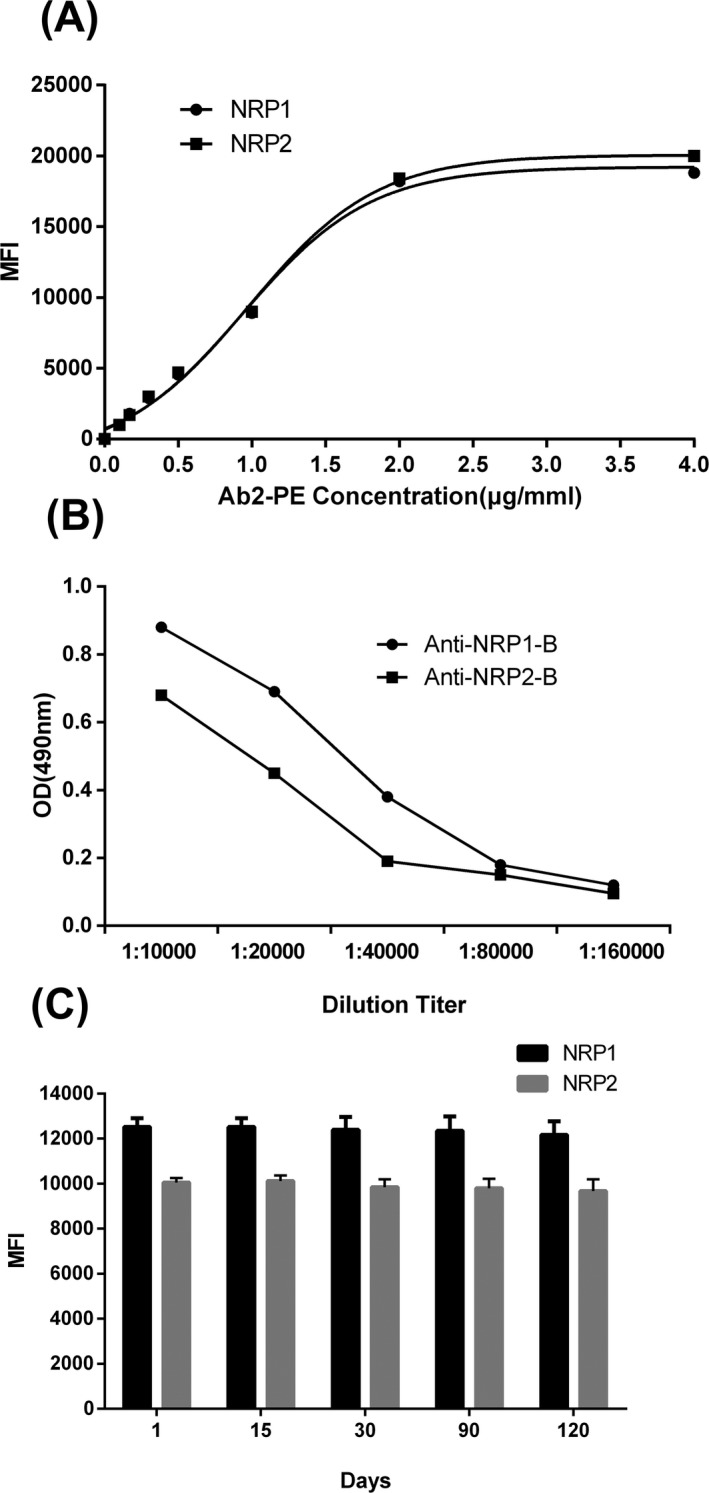
A, Analysis of magnetic beads coupling mAb efficiency. B, Titer analysis of antibody biotinylation. C, Influence of storage time on the performance of detection system

The magnetic beads coupled to mAb were confirmed by using a phycoerythrin‐conjugated antibody (IgG‐PE) directed against the Fc region of the respective coupled antibodies followed by measuring the mean fluorescence intensity (MFI) on the Luminex 200 platform. As shown in Figure 1A, with the increase in IgG‐PE concentration, the MFI value increased gradually and reached a plateau at 4 μg/mL. Thus, Nrp1 and Nrp2 mAbs had good coupling to the magnetic beads with no effect on their biological activities.

### 3.2 Identification of antibody biotinylation

The titer test of biotinylated antibodies was validated by direct ELISA. As shown in Figure 1B, with the increasing dilution ratio of biotinylated Nrp1 and Nrp2 mAbs, the OD490 nm values gradually reduced. Thus, the biotinylated Nrp1 and Nrp2 mAbs were stable and had high affinities, allowing their use in subsequent experiments.

### 3.3 Assessment of cross‐reactivity

Tests with irrelevant antigen standards showed far less fluorescence intensity than tests with relevant antigen standards, which indicated no cross‐reactivity in whole reaction system and that the prepared antibodies had high specificities (Table 3).

**Table 3:** Analysis of a cross‐reactivity between paired antibodies

Antigen	MFI		
	1	20	200 (ng/mL)
Sema3F	2	4	5
VEGF	2	4	*6*
CD34	2	3	4
NRP1	226	2346	18 660
NRP2	40	324	1944

MFI, mean fluorescence intensity.

### 3.4 Assay reproducibility

The MFI values obtained indicated reproducible results. Intra‐assay variances within a batch ranged from 1.0% to 2.6% for Nrp1 and from 2.9% to 4.0% for Nrp2. In addition, interassay variances between batches ranged from 1.5% to 6.4% for Nrp1 and from 4.2% to 8.1% for Nrp2, which were acceptable (Table 4).

**Table 4:** Determination of the coefficient of variation in batches **(**A**)** and between batches **(**B**)**

Standards (ng/mL)	NRP1		NRP2	
	MFI ± SD	CV (%)	MFI ± SD	CV (%)
(A)				
1	226 ± 4	1.8	40 ± 1.6	4
20	2346 ± 60	2.6	324 ± 12	3.7
200	18666 ± 186	1.0	1944 ± 56	2.9
(B)				
1	220 ± 14	6.4	42 ± 3.4	8.1
20	2230 ± 80	3.6	346 ± 16	4.6
200	18540 ± 276	1.5	2040 ± 86	4.2

MFI, mean fluorescence intensity.

### 3.5 Spike recovery

As shown in Table 5, the results were satisfactory, with recoveries of 96.6%‐103.6% for Nrp1 and 95.6%‐102.3% for Nrp2.

**Table 5:** Analysis of the spike recovery results

Samples + standards (ng/mL)	NRP1			NRP2		
	MFI ± SD	CV (%)	Recovery (%)	MFI ± SD	CV (%)	Recovery (%)
1 + 1	340 ± 28	8.4	96.6	48 ± 5.2	10.8	95.6
10 + 10	2310 ± 129	5.6	103.6	354 ± 20	5.6	102.3
100 + 100	18444 ± 368	2.0	99.5	1968 ± 76	3.9	96.5

MFI, mean fluorescence intensity.

### 3.6 Stability

Antibody‐cross‐linked beads, antigen standards, and biotin‐labeled antibodies were kept at 4°C for different numbers of days (1, 15, 30, 90, and 120 days) and then measured. Little change in the MFI was found, verifying the good stability of the beads (Figure 1C).

### 3.7 Comparison between multiplex assays and single ELISA

**Figure 2:**
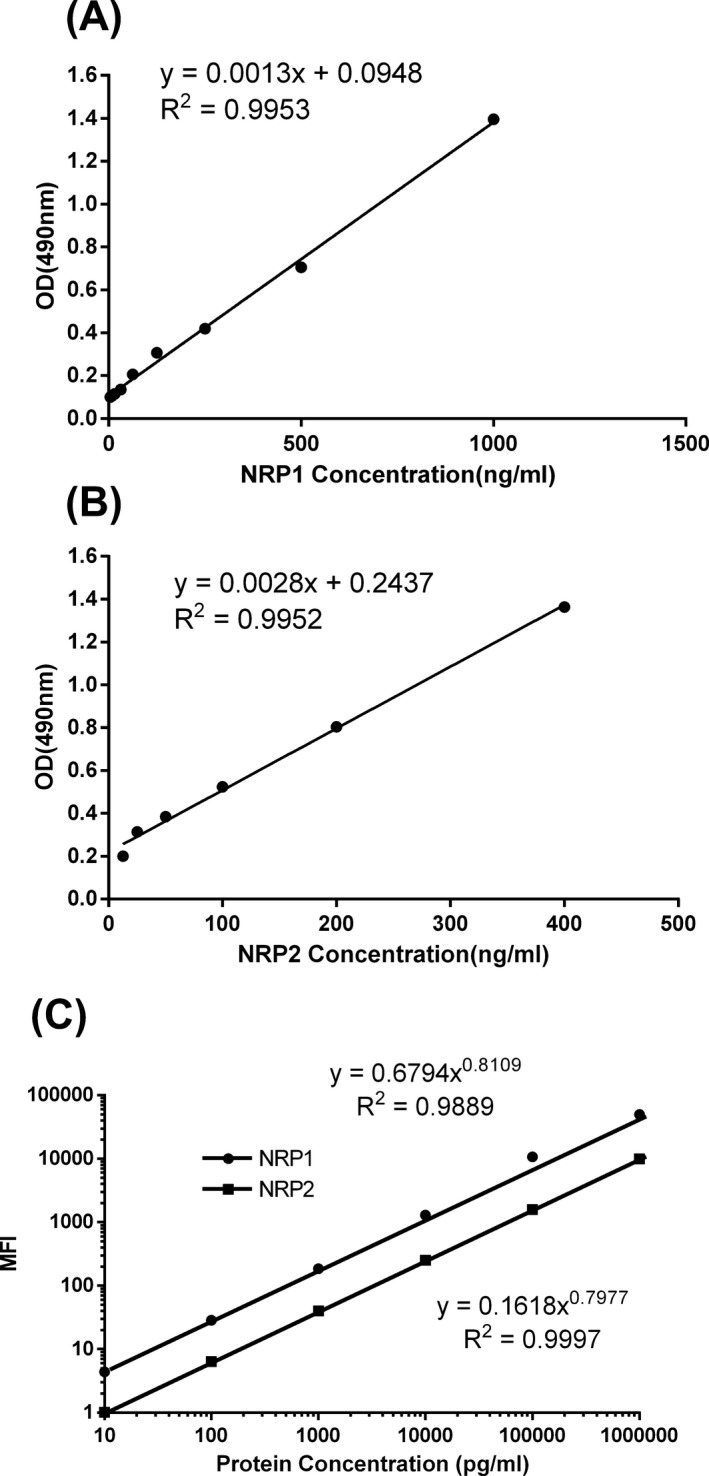
Sandwich ELISA standard curves of detection for Nrp1 (A) and Nrp2 (B). Standard curves of Nrp1 and Nrp2 from the duplex assay (C). Each standard curve shows optical density (OD) values and median fluorescence intensities (MFIs), respectively, which are presented as means and standard deviations obtained from three independent assays

The sandwich ELISA standard curves for Nrp1 and Nrp2 (Figure 2A,B) and the bead‐based assay standard curves for Nrp1 and Nrp2 (Figure 2C) are presented in Figure 2. The dynamic ranges of the sandwich ELISA standard curves are from 7 to 1000 ng/mL for Nrp1 and from 12.5 to 800 ng/mL for Nrp2, while the dynamic ranges of the bead‐based assay standard curves are from 10 to 100 000 pg/mL for Nrp1 and from 25 to 100 000 pg/mL for Nrp2. The sensitivity for Nrp1 and Nrp2 reached 10 pg/mL and 24.8 pg/mL, respectively. Thus, compared with traditional sandwich ELISA, the bead‐based assay has high sensitivity and a wide detection range.

### 3.8 Clinical applications

Upon detecting Nrp1 and Nrp2 in 100 normal human serum samples, the mean MFI value was 670 pg/mL with a maximum of 1200 pg/mL for Nrp1. For Nrp2, the mean MFI value was 463 pg/mL with a maximum of 850 pg/mL. Furthermore, we measured Nrp1 and Nrp2 in serum samples from 45 tumor patients. The concentrations of Nrp1 and Nrp2 in 43 of the tumor patient samples were within the ranges of those found in normal human serum. However, 2 of the tumor patient serum samples (30# and 31#) showed significantly higher concentrations of NRP1 and NRP2 than those of normal human serum. Serum sample 30# was from a colon cancer patient, and the serum concentrations of Nrp1 and Nrp2 were 15 000 and 9956 pg/mL, which were 22.4‐ and 21.5‐fold the mean values for serum from normal human samples. Serum sample 31# was from a gastric cancer patient, and the concentrations of Nrp1 and Nrp2 in the serum were 8630 and 8009 pg/mL, which were 12.9‐ and 17.4‐fold the mean values for serum from normal human samples.

## 4 DISCUSSION

Nrps are upregulated in a variety of cancers and can be secreted into the blood, which provides a novel prospect for studying the levels of Nrps in tumor patients’ serum and their connection with tumor development. However, there are currently no reports in the literature on the quantitative detection of Nrps.

In this study, we were able to develop a bead‐based duplex assay for simultaneously detecting Nrp1 and Nrp2 in human blood samples with good assay sensitivity, specificity, and stability; a broad dynamic range, and low intra‐assay and interassay variances. Thus, this assay has good potential clinical applications. The xMAP‐based assay method has the advantage of high‐throughput detection, and multiple indicators can be simultaneously detected. The whole assay process requires only 3.5‐4 hours and 2‐3 μL of serum sample, and therefore, the assay time and the amount of sample and reagent are greatly reduced. Our report establishes the basis for further development of diagnostic reagents for physical examination and early detection of cancer. We expect that the xMAP‐based assay of Nrp1 and Nrp2 could be clinically applied in early‐stage cancer screening, tumor malignancy analysis, and patient prognosis assessment.

## CONFLICT OF INTEREST

The authors have no financial interests to disclose.
